# Cryptotanshinone Attenuated Pathological Cardiac Remodeling *In Vivo* and *In Vitro* Experiments

**DOI:** 10.1155/2023/4015199

**Published:** 2023-01-27

**Authors:** Wen-jing Li, Han Yan, Zi-ying Zhou, Nan Zhang, Wen Ding, Hai-han Liao, Qi-zhu Tang

**Affiliations:** ^1^Department of Cardiology, Renmin Hospital of Wuhan University, Wuhan, China; ^2^Cardiovascular Research Institute of Wuhan University, Wuhan, China; ^3^Hubei Key Laboratory of Metabolic and Chronic Diseases, Wuhan, China; ^4^Department of Cardiac Function, Renmin Hospital of Wuhan University, Wuhan, China

## Abstract

**Objective:**

Cardiac remodeling has been demonstrated to be the early stage and common pathway for various types of cardiomyopathy, but no specific treatment has been suggested to prevent its development and progress. This study was aimed at assessing whether Cryptotanshinone (CTS) treatment could effectively attenuate cardiac remodeling *in vivo* and *in vitro*.

**Methods:**

Aortic banding (AB) surgery was performed to establish a pressure-overload-induced mouse cardiac remodeling model. Echocardiography and pressure-volume proof were used to examine mouse cardiac function. Hematoxylin and eosin (HE) and Picro-Sirius Red (PSR) staining were used to assess cardiac remodeling in vivo. Mouse hearts were collected to analysis signaling pathway and cardiac remodeling markers, respectively. Furthermore, neonatal rat cardiomyocyte (NRCMs) and cardiac fibroblast (CF) were isolated to investigate the roles and mechanisms of CTS treatment *in vitro*.

**Results:**

CTS administration significantly alleviated pressure-overload-induced mouse cardiac dysfunction, inhibited cardiac hypertrophy, and reduced cardiac fibrosis. Mechanically, CTS treatment significantly inhibited the STAT3 and TGF-*β*/SMAD3 signaling pathways. In vitro experiments, CTS treatment markedly inhibited AngII-induced cardiomyocyte hypertrophy and TGF-*β*-induced myofibroblast activation via inhibiting STAT3 phosphorylation and its nuclear translocation. Finally, CTS treatment could not protect against pressure overload-induced mouse cardiac remodeling after adenovirus-associated virus (AAV)9-mediated STAT3 overexpression in mouse heart.

**Conclusion:**

CTS treatment might attenuate pathological cardiac remodeling via inhibiting STAT3-dependent pathway.

## 1. Introduction

Pathological cardiac remodeling is characterized with structural and functional changes when the heart suffers from internal or external cardiovascular damage or sustained neurohumoral stimulation [1]. Pathological cardiac remodeling inevitably contributed to cardiac dysfunction and occurrence of heart failure (HF) [1]. HF has become one of the most burdensome ailments with approximately 23 million people worldwide [2]. Although the treatment strategies have made a breakthrough, the morbidity and mortality of HF remain very high [2]. Preventing or reversing cardiac remodeling has been suggested to be a potential strategy for protecting against HF. Activation of some signaling pathways including PI3K/AKT, MAPKs, JAK/STAT, and TGF-*β*/SMAD has been suggested to involve the development and progress of pathological myocardial remodeling. Targeting these activated pathways by pharmacological or genetic interference has been considered a potential strategy for slowing the progression of pathological myocardial remodeling and HF caused by various cardiovascular diseases.

**Figure 1:**
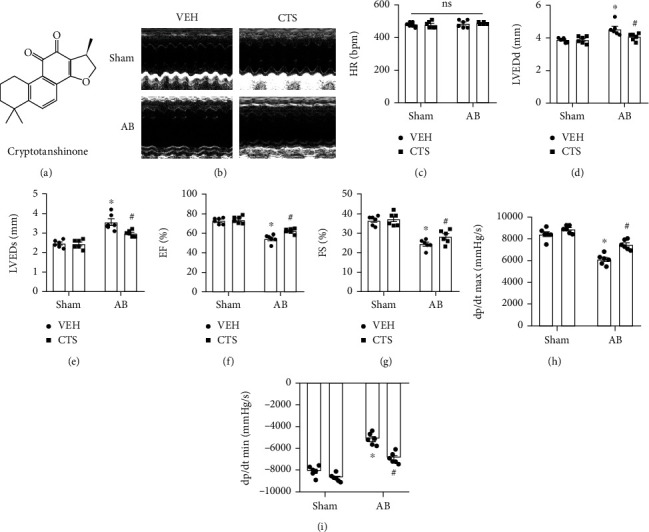
CTS treatment improved pressure overload-induced cardiac dysfunction. (a) The molecular structure of Cryptotanshinone (CTS); (b) the representative pictures of echocardiography; (c) heart rates (HR); (d) left ventricular end-diastolic diameters (LVEDd); (e) left ventricular end-systolic diameters (LVEDs); (f) left ventricle ejection fraction (EF); (g) shorting fraction (FS) and pressure-volume loop analysis presented: (h) maximal rate of pressure development (dp/dt max) and (i) minimal rate of pressure decay (dp/dt min); *n* = 6 in sham group and *n* = 6 in AB group. Data presented as the mean ± S.E. One-way ANOVA followed by Tukey's post hoc test was used for examining the significance difference among different groups. ^∗^Compared with the sham group (*p* < 0.05); ^#^compared with the AB+VEH group (*p* < 0.05).

Danshen (*Salvia miltiorrhiza Bunge*), a traditional herbal medicine, has been used for healthcare in promoting blood circulation, treating body pain, and improving body immunity in Asia especially in China, Korea, and Japan for over two millennia [3]. About 201 compounds have been extracted from danshen [3]. These compounds are divided into three groups according to molecular structure, including lipophilic diterpenoids, water-soluble phenolic acids, and others [3]. Cryptotanshinone (CTS) is extracted from danshen, belongs to lipophilic diterpenoids, and is an orally bioactive herbal compound [3, 4]. Its molecular structure is shown in Figure 1(a). CTS has been demonstrated to inhibit bladder cancer cell proliferation via downregulating PI3K/AKT/mTOR and NF-*κ*B signaling pathways [5]. CTS dose-dependently depressed tongue squamous cell carcinoma (TSCC) migration and proliferation; mechanistically, CTS inhibited the expression of p-STAT3 in TSCC [6]. CTS administration significantly decreased airway hyperresponsiveness and reduced inflammation responsiveness via modulating p38 MAPK phosphorylation and NF-*κ*B signaling pathways [7]. CTS administration by oral gavage in Sprague-Dawley rats effectively attenuated bleomycin-induced lung fibrosis via depressing phosphorylation of SMAD2/3 and STAT3 [8]. CTS treatment also inhibited SMAD2/3 and STAT3 phosphorylation in TGF-*β*1-treated human fetal lung fibroblasts [8]. These studies implied that CTS could exert various pharmacological activities, including anti-inflammation, antioxidative stress, and antitumor via regulating PI3K/AKT, MAPKs, TGF-*β*/SMAD, and STAT3 signaling pathways, which have been demonstrated to be involved in regulating pathological cardiac remodeling [1].

CTS has also been emerging to be a potential treatment strategy for treating cardiovascular disease. CTS treatment could markedly reduce atherosclerotic plaque formation and enhance plaque stability in ApoE-/- mice via inhibiting reactive oxygen species (ROS) production and NF-*κ*B activation [4]. CTS treatment significantly depressed doxorubicin induced ROS production and cardiomyocyte apoptosis via suppressing p53 signaling pathway [9]. In addition, CTS administration could effectively attenuate diabetic cardiomyopathy associated cardiac fibrosis by reducing matrix metalloproteinase-9 (MMP-9) and connective tissue growth factor (CTGF) production via inhibiting STAT3 activity [10]. However, its roles and mechanisms in pressure overload-induced cardiac remodeling have not yet been reported. Therefore, this study is mainly aimed at clarifying the curative properties of CTS on pressure overload-induced pathological myocardial remodeling *in vivo*. Moreover, this study also investigated the protective roles and mechanisms in angiotensin II- (AngII-) treated neonatal rat cardiomyocytes (NRCMs) and TGF*β*-treated neonatal rat cardiac fibroblast (CF) remodeling *in vitro*.

## 2. Material and Methods

### 2.1. Chemicals and Agents

Cryptotanshinone (purity ≥ 99%) was purchased from Shanghai Winherb Medical Technology Co, Ltd (35825-7-1). AngII was obtained from ENZO (ALX-151-039-M025). TGF-*β* was purchased from Abcam (ab50036). Adenovirus-mediated STAT3 (Ad-STAT3) and adenovirus-associated virus (AAV)9-mediated STAT3 (AAV9-STAT3) were obtained from WZ Biosciences Inc.

### 2.2. Mice and Aortic Banding Model

Male C57BL/6J mice (8-10 weeks, 24.5-25.5 g) were purchased from the Institute of Laboratory Animal Science (CAMS&PUMS, Beijing, China). All animals were feed a chow diet in a 12-hour light/12-hour dark environment at 22-24°C and 50-60% humidity in the Cardiovascular Research Institute of Wuhan University (Wuhan, China). All the animal experimental procedures were conducted in accordance with the National Institute of Health (NIH) Guide for the Care and Use of Laboratory Animals and were approved by the Guidelines of the Animal Care and Use Committee of Renmin Hospital of Wuhan University (approval no. 20180303).

The mouse model of cardiac remodeling via Aortic Banding (AB) surgery was established as previously described [11]. Briefly, mice were weighed and anesthetized with 3% pentobarbital sodium (50 mg/kg) by intraperitoneal injection. And then, isolate the thoracic aorta of mice and a 7-0 surgical suture was used to ligate a 27G needle to the aorta. After ligation, the blunt needle was pulled out, resulting a 70% contraction of aorta. Sham group mice underwent the same operation but without ligation.

All animal studies were performed blindly. After AB surgery, the mice were divided into four groups randomly: saline group (VEH), CTS group, AB group, and AB+CTS group (*n* = 10 in the sham group, *n* = 10 in the AB group). Three days after AB surgery, mice were administrated with CTS for 4 weeks (50 mg/kg, gastric perfusion) [12]. At the end of the treatment, the surviving mice were used for accessing cardiac function. And mice were euthanized with an overdose of sodium pentobarbital (200 mg/kg, intraperitoneal injection). Hearts were collected for other experiments.

### 2.3. Adenovirus-Associated Virus 9 Vector

AAV9-STAT3 was obtained from WZ Biosciences Inc. as previously described [13]. Briefly, plasmid with the cytomegalovirus (CMV) promoter directing expression of human STAT3 was used to product AAV9-STAT3. And then, AAV9 vector was packaged in HEK293 cells by the double or triple transfection method. Ammonium sulfate fractionation and iodixanol gradient centrifugation were used to purify the virus. In the end, titers of the AAV9 vector were determined by quantitative real-time PCR (qRT-PCR) [14]. The green fluorescent protein- (GFP-) expressing vector (AAV9-GFP) was used as control.

In vivo, 60-80 *μ*l AAV9-STAT3 (5.0-6.5 × 10^13^ genome copies(GC)/ml) was injected into the retroorbital venous plexus of the mice to overexpress STAT3 [15]. After one week, the mice underwent AB surgery or CTS administration [13]. Four weeks after AB surgery or CTS administration, mouse hearts were excised and total genomic DNA was extracted. qRT-PCR was used to detected AAV9 vector genome copy numbers.

### 2.4. Assessing Cardiac Function

Four weeks after the administration of CTS, mice were anesthetized with 1.5% isoflurane to detect the cardiac function via echocardiogram and pressure-volume loop analysis. The detailed protocols were established as previously described [11]. Echocardiogram was performed by using a Mylab 30CV ultrasound system (Biosound Esaote Inc.) equipped with a 10 MHz ultrasound transducer. Pressure-volume loop analysis was performed by using a microtip transducer catheter (SPR-839, Millar Instruments, Houston, TX, USA) to insert into the right carotid artery and advance into the left ventricle. The pressure and heart rate signal were recorded by a Millar Pressure-Volume System (MPVS-400, Instruments).

### 2.5. Histological Analysis and Immunohistochemistry

Mouse hearts were excised and put into 10% KCl to arrest in diastole stage and then were transferred into 10% formalin for fixing 24 hours. After being photographed by a camera (Nikon D700), mouse hearts were embedded in paraffin and cut into sections of thickness of 5 *μ*m. After dewaxing and rehydrating, mouse heart sections were used hematoxylin and eosin (HE) staining, to assess the cross-sectional area of cardiomyocytes, and Picro-Sirius Red (PSR) staining to assess the fibrosis area in perivascular and cardiac interstitium. The detailed methods were performed as previously described [16–18]. Quantitative digital image analysis system (Image-Pro Plus 6.0) was used for image analysis. About 200 left ventricle myocytes from different heart sections were traced for assessing cardiomyocyte hypertrophy in vivo.

### 2.6. Cell Culture and Treatment

Neonatal rats (1 to 2 days, Sprague-Dawley rats) were used for isolating cardiomyocytes and cardiac fibroblast. The detailed protocol was established as previously described [19]. Briefly, the heart tissue in the left ventricle was cut into 1 mm^3^ fragments in DMEM/F12 medium. Then, the collected tissue fragments were digested with 0.125% Trypsin (Gibco, 25200) at 37°C for 15 min × 5 times. Centrifuge and collect cell suspension to collect the cellular precipitation, which was resuspended in DMEM/F12 medium supplied with 15% fetal bovine serum (FBS, HyClone). A differential attachment technique was used to isolate the NRCMs and CFs. After 90 min of differential attachment culture, the CFs were attached to the bottom of culture dish while the NRCMs existed in the culture medium. The NRCMs were counted and cultured in a 6-well plate with cell density of 5 × 10^6^ and treated with AngII (1 *μ*M) or PBS in the presence of different concentrations of CTS (0, 3, 6, and 12 *μ*M) for 12 h. The CFs were passaged to the second generation and then were counted and cultured in a 6-well plate with a cell density of 2 × 10^6^ and treated with TGF-*β* (10 *μ*M) or PBS in the presence of different concentrations of CTS (0, 3, 6, and 12 *μ*M) for 12 h. The dose of AngII (1 *μ*M) and TGF-*β*(10 *μ*M) was performed as previously described [20, 21]. Western blot was performed to assess the effect of CTS.

Ad-STAT3 was used to overexpress STAT3. NRCMs were transfected with Ad-STAT3 or Ad-GFP for 24 h and then were incubated with CTS or AngII for another 12 hours before collecting for total protein extraction.

### 2.7. Immunofluorescence Analysis

Immunofluorescence staining was performed to examine cardiomyocyte hypertrophy and myofibroblast activation. Cells were seeded in 24 wells. NRCMs were treated with AngII (1 *μ*M) or PBS in the presence or absence of CTS (12 *μ*M) for 12 h. CFs were stimulated with TGF-*β* (10 *μ*M) or PBS in the presence or absence of CTS (12 *μ*M) for 48 h. After that, cells were washed with PBS and fixed with 10% paraformaldehyde. After being permeabilized with 0.1% Triton X-100, NRCMs and CFs were incubated with *α*-actin (1 : 100) or *α*-SMA (1 : 100), respectively, overnight. The next day, the cells were incubated with the fluorescent secondary antibody (Alexa Fluor ®, Invitrogen) and then were stained with DAPI (Invitrogen) and covered with glass slides. The fluorescent pictures were captured with a fluorescence microscope and analyzed by Image-Pro Plus 6.0.

### 2.8. Examining Cell Viability and Proliferation

Enhanced Cell Counting Kit-8 (CCK-8, Beyotime, Shanghai, China) was used to detect the cytotoxicity of CTS and fibroblast proliferation according to the manufacturer's protocol. For testing CTS's cytotoxicity, NRCMs were seeded in 96-well plates at a density of 4 × 10^4^ cells/well. Five concentrations of CTS (0, 3, 6, 12, and 24 *μ*M) were added into the plate [22]. After incubating with 48 hours, the cell viability was examined by CCK-8. The concentration of 12 *μ*M was used for further experiment.

For testing cardiac fibroblast proliferation, CFs were seeded in 96-well plates at a density of 4 × 10^4^ cells/well. CTS and TGF-*β* were added into the plate according to the discretion in the figure legend for 48 hours; the cell proliferation was examined by CCK-8.

A microplate reader (Synergy HT) was used to read the absorbance value at 450 nm. Each assay was repeated in triplicate.

### 2.9. Western Blot Analysis

Heart tissues (20-30 mg), collected NRCMs, or CFs were lysed in RIPA buffer (Servicebio, G2002) supplemented with Protease Inhibitor Cocktail Tablets complete (Roche) and Phosphatase Inhibitor Cocktail Tablets Pho-STOP(Roche). BCA Protein Assay Kit (Thermo) was used to quantify the extracted total protein from heart tissue or cells. Total protein (30 *μ*g) was separated by SDS-PAGE electrophoresis followed by transferring to polyvinylidene fluoride (PVDF) membranes. Following blockade with TBS containing 5% skim milk, membranes were incubated with primary antibodies overnight at 4°C. The next day, the membranes were incubated with corresponding horseradish peroxidase-conjugated secondary antibodies (Abbkine). ChemiDoc™ Imaging System (Bio-Rad) was used to analysis the blots. Immunoblots were quantified using Image Lab™ software (version 6.0). These following primary antibodies were used in this study: GAPDH (CST, 2118), Phospho-STAT3 (Tyr705) (CST, 9138), STAT3(CST, 9139), Histone H3 (ABCAM, ab5176), p-SMAD2 (Ser465/467) (CST, 3180), SMAD2 (CST, 3130), p-SMAD1/5(Ser463/465) (CST, 9516), SMAD1/5 (SANTA, sc6201), p-SMAD3 (Ser423/S425) (ABCAM,ab52903), SMAD3 (CST, 9513), BAX (CST, 2772), BCL2 (CST, 2870), TGF-*β* (Abcam, ab66403), FN (SANTA, sc-6952), *α*-SMA (ABCAM, ab5694), ANP (SANTA, sc-515701), *β*-MHC (SANTA, sc-530090), and BNP (ABCAM, ab239510).

### 2.10. Quantitative Real-Time Reverse Transcriptase-PCR (qRT-PCR)

Total RNA was extracted using the TRIzol reagent (Inivtrogen, USA). cDNA was synthesized from 2 *μ*g of total RNA with the Transcriptor First Strand cDNA Synthesis Kit (Roche,04896866001). LightCycler 480 SYBR Green 1 Master Mix was used to detect the PCR amplification LightCycler 480 (Roche). The mRNA expression of target genes was normalized to GAPDH before calculating the relative fold change. Primers used for the experiment are shown in Table 1.

### 2.11. Statistical Analysis

Results are presented as the mean ± SEM. One-way ANOVA followed by Tukey's post hoc test was used for examining the significance difference among different groups. All data were analyzed by using SPSS 18.0 (SPSS Inc.). *p* < 0.05 was considered statistically significant.

## 3. Results

### 3.1. CTS Administration Improved Pressure Overload Induced Mouse Cardiac Dysfunction

Mouse were subjected to AB surgery to establish pressure overload-induced cardiac remodeling model *in vivo* and then were administrated with CTS (50 mg/kg/d, by gastric perfusion) for 4 weeks after 3 days of AB surgery. Echocardiography was performed to assess mouse cardiac function (Figure 1(b)). The heart rate among different groups showed nonsignificant difference (Figure 1(c)). Mice suffered significantly cardiac dysfunction after AB surgery evidenced by increased LVEDd and LVEDs and decreased EF and FS (Figures 1(d)–1(g)). CTS treatment significantly reduced LVEDd and LVEDs and restored EF and FS in AB+CTS group compared with the AB+VEH group (Figures 1(d)–1(g)). Catheter-based pressure-volume loop analysis demonstrated that AB surgery caused a significant increase of dp/dt max and dp/dt min in AB+VEH group compared with the sham group (Figures 1(h) and 1(i)); however, CTS administration markedly decreased dp/dt max and dp/dt min in the AB+CTS group compared with the AB+VEH group (Figures 1(h) and 1(i)). These data exhibited that CTS treatment could improve pressure overload-induced mouse cardiac dysfunction.

### 3.2. CTS Treatment Attenuated Cardiac Hypertrophy

**Figure 2:**
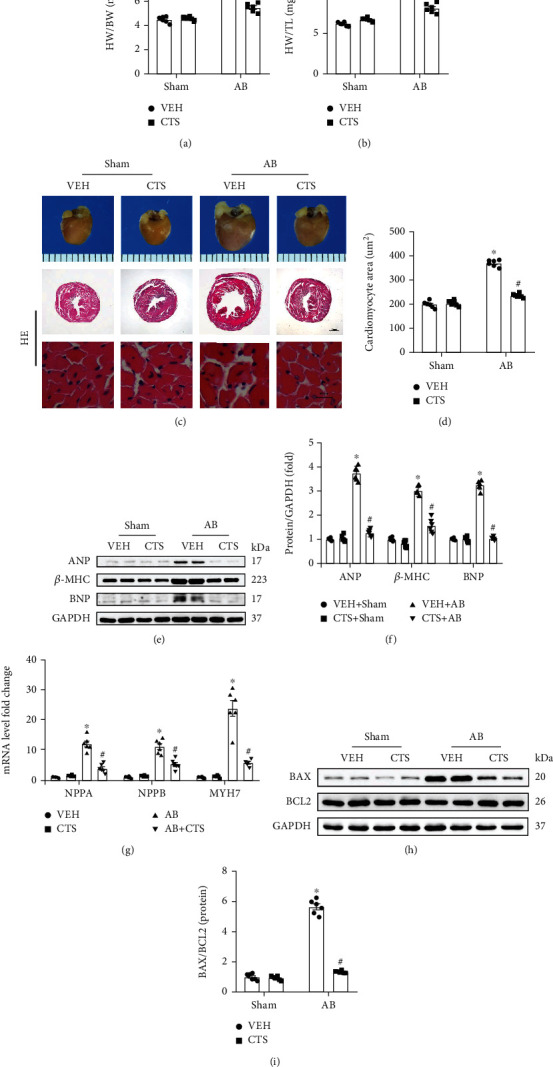
CTS treatment attenuated mouse cardiac hypertrophy; (a) heart weight/body weight (HW/BW) (*n* = 6 in sham group, *n* = 6 in AB group); (b) heart weight/tibia length (HW/TL) (*n* = 6 in sham group, *n* = 6 in AB group); (c) the morphological changes and HE staining for mouse heart (*n* = 6); (d) calculating the cardiomyocyte surface area according to the HE staining (*n* = 6); (e) representative western blots for ANP, *β*-MHC and BNP, and relative quantification of (f) ANP/GAPDH, *β*-MHC/GAPDH, and BNP/GAPDH; (g) RT-PCR examined the mRNA expression of NPPA, NPPB and MYH7 (*n* = 6). (h) Representative Western blots for BAX, BCL2, and GAPDH; (i) relative quantification of BAX/BCL2. GAPDH was used for loading control. The protein and mRNA expression were normalized to GAPDH at its protein or mRNA expression, respectively, before the relative quantification. Data presented as mean ± S.E. One-way ANOVA followed by Tukey's post hoc test was used for examining the significance difference among different groups. ^∗^Compared with the sham group (*p* < 0.05); ^#^compared with the AB+VEH group (*p* < 0.05).

Pressure overload significantly contributed to cardiac hypertrophy exhibited by increased heart weight/body weight (HW/BW) and heart weight/tibia length (HW/TL), and raised cardiac mass and cardiomyocyte cross-sectional area (CSA) in the AB+VEH group compared with the sham group (Figures 2(a)–2(d)). CTS treatment significantly decreased HW/BW, HW/TL, cardiac mass, and CSA in the AB+CTS group compared with the AB+VEH group (Figures 2(a)–2(d)). The protein expression of ANP, BNP, and *β*-MHC was significantly increased in the AB+VEH group compared with the sham group; however, CTS treatment significantly inhibited ANP, BNP, and *β*-MHC expression (Figures 2(e) and 2(f)). Moreover, pressure overload significantly induced mRNA overproduction of NPPA, NPPB, and MYH7 in the AB+VEH group, which could be effectively inhibited by CTS treatment in the AB+VEH group compared with the AB+VEH group (Figure 2(g)). Apoptosis is a predominant feature in pathological cardiac hypertrophy and related to the progression of cardiac hypertrophy [23, 24]. Therefore, we also assessed the expression of proapoptotic and antiapoptotic proteins. Pressure overload promoted the BAX overexpression but depressed BCL2 expression in the AB group compared with the sham group (Figures 2(h) and 2(i)). Contrarily, CTS treatment significantly inhibited BAX expression and reversed BCL2 expression in the AB+CTS group compared with the AB+VEH group (Figures 2(h) and 2(i)). These data exhibited that CTS administration significantly attenuate pressure overload-induced mouse cardiac hypertrophy.

### 3.3. CTS Treatment Alleviated Cardiac Fibrosis

**Figure 3:**
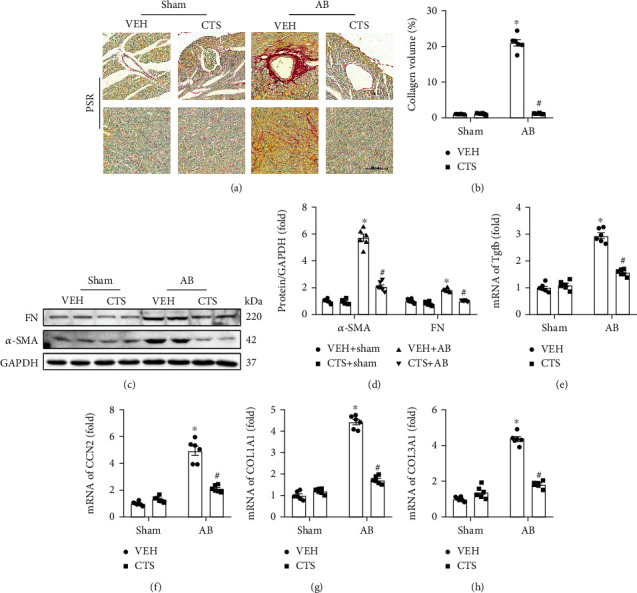
CTS treatment attenuated mouse cardiac fibrosis. (a) PSR staining to detected interstitial and perivascular fibrosis in the left ventricle (*n* = 6); (b) calculation of the PSR-positive area in the left ventricle according to PSR staining (*n* = 6); (c) representative Western blots for FN, *α*-SMA, and GAPDH (*n* = 6); (d) relative quantification of FN/GAPDH and *α*-SMA/GAPDH (*n* = 6); RT-PCR examined the mRNA expression of (e) Tgfb, (f) CCN2, (g) COL1A1, and (h) COL3A1 in indicated groups (*n* = 6). GAPDH was used for loading control. The protein and mRNA expression was normalized to GAPDH at its protein or mRNA expression, respectively, before the relative quantification. Data presented as the mean ± S.E. One-way ANOVA followed by Tukey's post hoc test was used for examining the significance difference among different groups. ^∗^Compared with the sham group (*p* < 0.05); ^#^compared with the AB+VEH group (*p* < 0.05).

Cardiac fibrosis is an essential character of pressure overload-induced cardiac remodeling. PSR staining showed that 4 weeks of pressure overload-induced markedly interstitial and perivascular fibrosis in the AB+VEH group compared with the sham group (Figures 3(a) and 3(b)). CTS treatment significantly alleviated interstitial and perivascular fibrosis in the AB+CTS group compared with the AB+VEH group (Figures 3(a) and 3(b)). Western blot examination demonstrated that fibronectin (FN) and *α*-smooth muscle actin (*α*-SMA) were significantly upregulated in the AB+VEH group compared with the sham group (Figures 3(c) and 3(d)); however, CTS treatment significantly decreased FN and *α*-SMA expression in the AB+CTS group compared with the AB+VEH group (Figures 3(c) and 3(d)). RT-PCR was performed to further examine mRNA expression of the fibrosis-associated biomarkers including Tgfb, CCN2, COL1A1, and COL3A1, all of which were significantly elevated in the AB+VEH group compared with the sham group (Figures 3(e)–3(h)), but CTS treatment could effectively inhibit the expression of these fibrosis-associated biomarkers in the AB+CTS group compared with the AB+VEH group (Figures 3(e)–3(h)).

### 3.4. CTS Treatment Blocked STAT3 and TGF-*β*/SMAD Signaling Pathway

**Figure 4:**
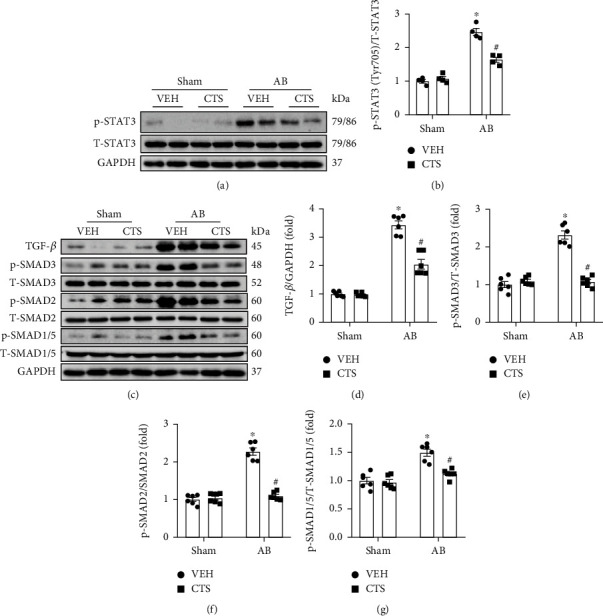
CTS treatment inhibited STAT3 and TGF-*β*/SMAD pathways. (a) Representative Western blots of p-STAT3, T-STAT3, and GAPDH; (b) relative quantification of p-STAT3/T-STAT3; (c) representative Western blots of TGF-*β*, p-SMAD3, T-SMAD3, p-SMAD2, T-SMAD2, p-SMAD1/5, T-SMAD1/5, and GAPDH; relative quantification of (f) TGF-*β*/GAPDH, (d) p-SMAD3/T-SMAD3, (e) p-SMAD2/T-SMAD2, and (f) p-SMAD1/5/T-SMAD1/5. (g) All proteins were normalized to corresponding total protein and then normalized to GAPDH before relative quantification (*n* = 6). Data presented as the mean ± S.E. One-way ANOVA followed by Tukey's post hoc test was used for examining the significance difference among different groups. ^∗^Compared with the sham group (*p* < 0.05); ^#^compared with the AB+VEH group (*p* < 0.05).

Previous published studies indicated that CTS might be involved in regulating several signaling pathways including MAPKs, AKT, and STAT3 phosphorylation. We screened the phosphorylation state of these implying signaling pathways. This study showed that CTS administration has nonsignificant effect on MAPKs (p38, ERK1/2, and JNK1/2) and PI3K/AKT signaling pathways (Supplementary figure 1, D-F). Pressure overload significantly activated JAK/STAT3 signaling pathway, evidenced by significantly increased phosphorylation of JAK1, JAK2, and STAT3 in the AB+VEH group compared with the sham group (Supplementary figure 1, A-C, Figures 4(a) and 4(b)). However, CTS treatment significantly inhibited STAT3 phosphorylation but showed nonsignificant effect on JAK1 and JAK2 phosphorylation in the AB+CTS group compared with the AB+VEH group (Supplementary figure 1, A-C, Figures 4(a) and 4(b)). Pressure overload also significantly enhanced TGF-*β* expression and phosphorylation of SMAD3, SMAD2, and SMAD1/5 (Figures 4(c)–4(g)), which indicated that pressure overload induced a significant activation of fibrosis associated signaling pathways. CTS treatment could significantly depress TGF-*β* expression and phosphorylation of SMAD3, SMAD2, and SMAD1/5 in the AB+CTS group compared with the AB+VEH group (Figures 4(c)–4(g)).

### 3.5. CTS Suppressed AngII-Induced NRCM Hypertrophy via Inhibiting STAT3 Phosphorylation

**Figure 5:**
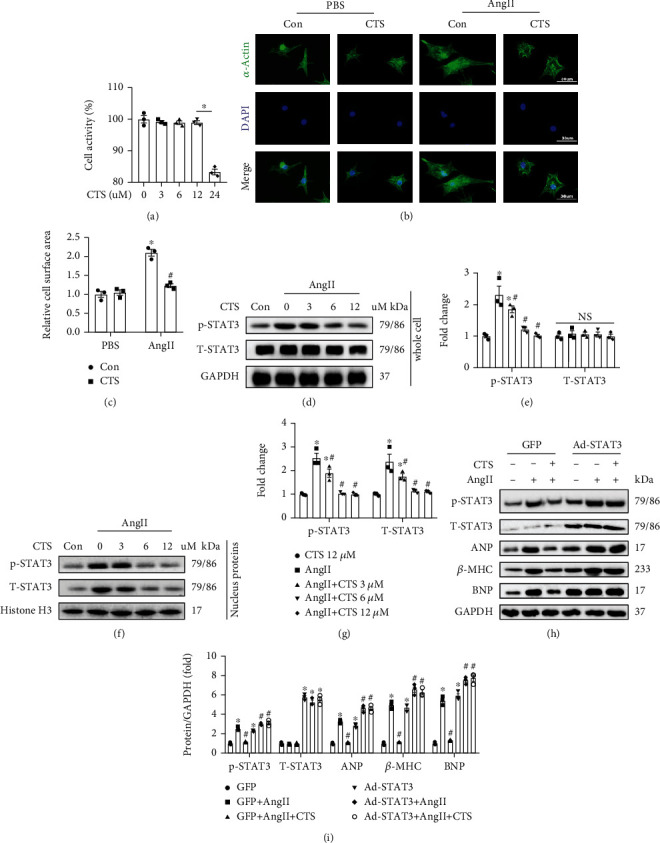
CTS treatment inhibited AngII induced cardiomyocyte hypertrophy in vitro. (a) CCK-8 was used to determine the cell viability after treating different concentration of CTS (0, 3, 6, 12, and 24 *μ*M) for 48 hours; (b) representative fluorescent pictures for *α*-actin and NRCMs were treated with AngII (1 *μ*M) or CTS for 48 hours; (c) calculating the CSA according to the fluorescent pictures, about 100 NRCMs were used for calculating; (d) representative Western blots of p-STAT3, T-STAT3, and GAPDH; NRCMs were incubated with AngII or CTS (3, 6, or 12 *μ*M) for 12 h; (e) relative quantification of p-STAT3/T-STAT3 and T-STAT 3; (f) representative Western blots of p-STAT3, T-STAT3, and Histone H3; NRCMs were incubated with AngII or CTS (3, 6, or 12 *μ*M) for 12 h, and then isolate the nucleus proteins from NRCMs for analysis; (g) relative quantification of p-STAT3/T-STAT3 and T-STAT 3; (h) representative Western blots of p-STAT3, T-STAT3, ANP, *β*-MHC, BNP, and GAPDH; NRCMs were transfected with adenovirus for mediating GFP or STAT3 overexpression during 24 h and then were incubated with CTS or AngII as indicated in picture for another 12 hours before collecting for total protein extraction; (i) relative quantification of p-STAT3/T-STAT3, T-STAT3, ANP, *β*-MHC, and BNP; all phosphorylated proteins were normalized to corresponding total protein and then normalized to GAPDH before relative quantification; cell experiments were repeated three times independently. ^∗^Compared with the PBS-treated group (*p* < 0.05); ^#^compared with the AngII-treated group (*p* < 0.05).

NRCMs were isolated and were treated with different concentrations of CTS (0, 3, 6, 12, and 24 *μ*M) for 48 hours (Figure 5(a)). Concentration of CTS (≤12 *μ*M) showed none significant effect for NRCM viability (Figure 5(a)). 12 *μ*M of CTS was used for the next experiments. AngII treatment caused significantly hypertrophy of NRCMs, which could be effectively blocked by CTS treatment for 24 h (Figures 5(b) and 5(c)). Mechanistically, AngII treatment significantly induced STAT3 hyperphosphorylation at 1, 4, 8, 12, and 24 h, respectively, compared with the nontreatment group (Supplementary figure 2, A and B). CTS treatment could effectively inhibit AngII-induced STAT3 phosphorylation in NRCMs (Figures 5(d) and 5(e)). The hyperphosphorylated STAT3 was mainly transferred into the nucleus (Figures 5(f) and 5(g)); however, CTS treatment markedly inhibited STAT3 phosphorylation and its nucleus accumulation (Figures 5(f) and 5(g)). AngII-mediated STAT3 hyperphosphorylation was accompanied with overproduction of ANP and *β*-MHC; however, CTS treatment could effectively inhibit STAT3 phosphorylation and decrease ANP and *β*-MHC expression (Figures 5(h) and 5(i)). Moreover, adenovirus-mediated STAT3 (Ad-STAT3) overexpression in NRCMs further exacerbated AngII-induced STAT3 hyperphosphorylation and overexpression of ANP and *β*-MHC. CTS treatment also could not prevent overproduction of ANP and *β*-MHC after Ad-STAT3 mediated STAT3 overexpression in NRCMs (Figures 5(h) and 5(i)).

### 3.6. CTS Treatment Prevented against TGF-*β* Induced Fibrosis In Vitro

**Figure 6:**
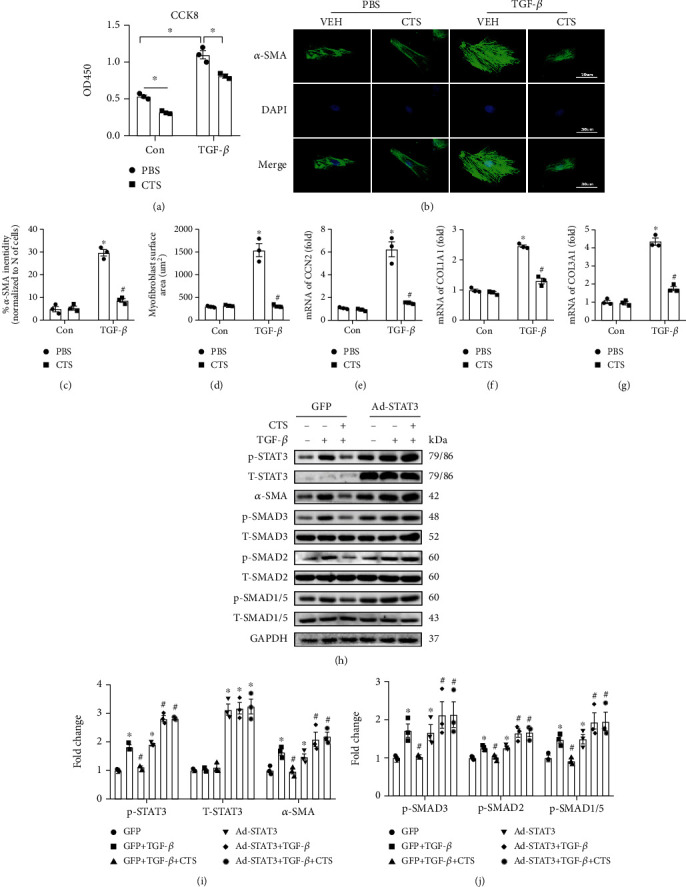
CTS treatment inhibited TGF-*β*-induced proliferation and myofibroblast activation. (a) CCK-8 determined the proliferation of isolated neonatal CFs and CFs was treated with PBS, TGF-*β* (10 *μ*M), or CTS (12 *μ*M) for 48 hours as indicated in the pictures; (b) immunofluorescence staining indicated that TGF-*β* treatment (10 *μ*M, 48 hours) induced *α*-SMA expression; CTS treatment (12 *μ*M) significantly inhibited TGF-*β* induced *α*-SMA expression; (c) assessing the *α*-SMA fluorescence intensity; (d) calculating the cell surface area (CSA), about 100 NRCMs were used for calculating CSA; relative quantification expression of mRNA of (e) CCN2, (f) COL1A1, and (g) COL3A1; CFs were incubated with CTS or TGF-*β* for 24 hours before collecting for RNA extraction; the mRNA expression of these target genes was normalized to GAPDH before relative quantification; (h) representative Western blots of p-STAT3, T-STAT3, *α*-SMA, p-SMAD3, T-SMAD3, p-SMAD2, T-SMAD2, p-SMAD1/5, T-SMAD1/5, and GAPDH; CFs were transfected with adenovirus for GFP or STAT3 overexpression during 24 h and then were incubated with CTS or TGF-*β* as indicated in picture for another 12 hours before collecting for total protein extraction; (i) relative quantification of p-STAT3/T-STAT3; (j) relative quantification of TGF-*β*, p-SMAD3/T-SMAD3, p-SMAD2/T-SMAD2, and p-SMAD1/5/T-SMAD1/5; all of these proteins were normalized to GAPDH before the relative quantification; cell experiments were repeated three times independently. ^∗^Compared with the PBS-treated group (*p* < 0.05); ^#^compared with the TGF-*β*-treated group (*p* < 0.05).

CFs isolated from neonatal rat hearts passaged with 3 generations were used for the following experiments. TGF-*β* treatment significantly promoted CF proliferation in the TGF*β*+PBS group compared with the PBS group (Figure 6(a)); however, CTS treatment significantly inhibited both base line and TGF-*β*-induced CF proliferation (Figure 6(a)). Immunofluorescence staining demonstrated that TGF-*β* treatment significantly increased cellular surface area (CSA) and promoted myofibroblast activation evidenced by enhanced *α*-SMA expression in the TGF-*β*+PBS group compared with the VEH+PBS group (Figures 6(b)–6(d)); however, CTS treatment markedly inhibited *α*-SMA expression and reduced the CSA in the TGF-*β*+CTS group compared with the TGF-*β*+PBS group (Figures 6(b)–6(d)). Meanwhile, RT-PCR analysis demonstrated that TGF-*β* treatment obviously induced mRNA expression of CCN2, COL1A1, and COL3A1 (Figures 6(e)–6(g)), all of which could be significantly inhibited by CTS treatment in the TGF-*β*+CTS group compared with the TGF-*β*+PBS group (Figures 6(e)–6(g)). Mechanistically, TGF-*β* treatment markedly enhanced the STAT3 phosphorylation (Figures 6(h)–6(j)), promoted *α*-SMA expression, and activated SMAD signaling pathway (Figures 6(h)–6(j)). CTS treatment markedly inhibited TGF-*β*-induced *α*-SMA expression and phosphorylation of STAT3, SMAD2, SMAD3, and SMAD1/5 (Figures 6(h)–6(j)). CTS treatment could no longer inhibit *α*-SMA expression and activation of SMAD signaling pathway after adenovirus mediated STAT3 overexpression (Figures 6(h)–6(j)).

### 3.7. CTS Treatment Could Not Protect against Pressure Overload-Induced Cardiac Hypertrophy after AAV9-Mediated STAT3 Overexpression in Mouse Heart

**Figure 7:**
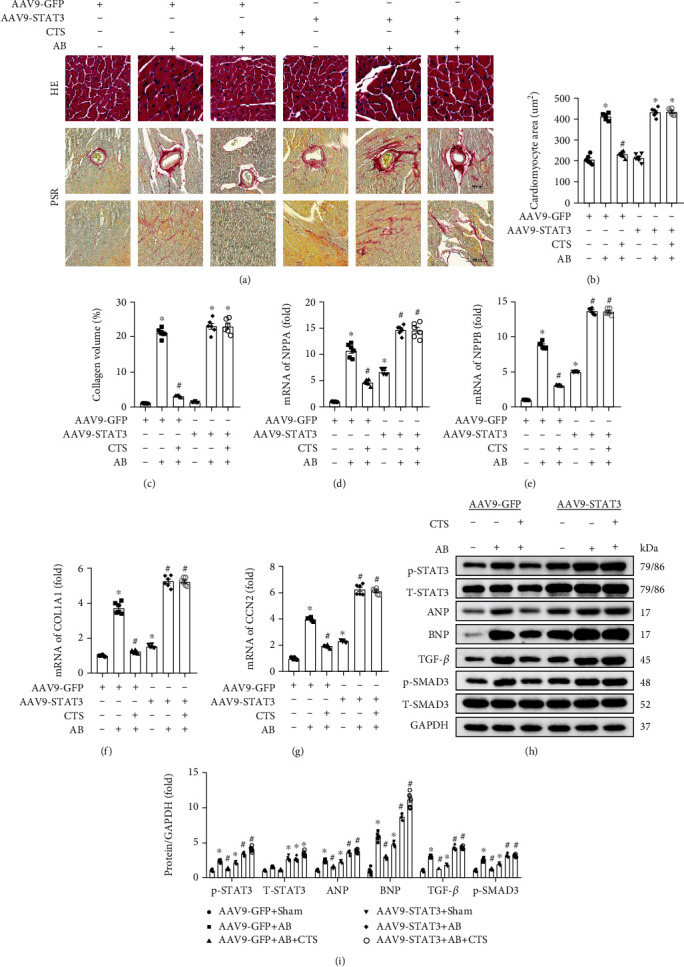
CTS treatment could not protect against cardiac hypertrophy after AAV9 mediated STAT3 overexpression. (a) Representative pictures of HE and PSR staining (*n* = 6); (b) calculating cell surface area (CSA) according to HE staining (*n* = 6); (c) calculating fibrosis area according to PSR staining (*n* = 6); relative quantification expression of mRNA of (d) NPPA, (e) NPPB, (f) COL1A1, and (g) CCN2 and the mRNA expression of these target genes were normalized to GAPDH before relative quantification (*n* = 6); (h) representative western blots of p-STAT3, T-STAT3, ANP, BNP, TGF-*β*, p-SMAD3, T-SMAD3, and GAPDH (*n* = 6); (i) relative quantification of p-STAT3, T-STAT3, ANP, BNP, TGF-*β*, and p-SMAD3/T-SMAD3 (*n* = 6); all of these proteins were normalized to GAPDH before the relative quantification; ^∗^Compared with the AAV9-GFP group (*p* < 0.05); ^#^compared with the AAV9-GFP group+AB group (*p* < 0.05).

CTS treatment could no longer protect against pressure overload-induced mouse cardiac hypertrophy and fibrosis after AAV9-mediated STAT3 overexpression. Nonsignificant difference could be observed in the CSA, interstitium, and perivascular fibrosis between the AB+CTS and AB+CTS+AAV9-STAT3 groups (Figures 7(a)–7(c)). There was also nonsignificant difference of hypertrophy and fibrosis-associated biomarkers including NPPA, NPPB, COL1A1, and CCN2 between the AB+CTS and AB+CTS+AAV9-STAT3 groups (Figures 7(d)–7(g)). Echocardiography demonstrated that CTS treatment could not reduce LVEDd and LVEDs and also could not restore LVEF and FS after AAV9-mediated STAT3 overexpression (Supplementary figure 3, A-D). Western blot analysis presented that AAV9-mediated STAT3 overexpression exacerbated the phosphorylation of STAT3 and Smad3 and enhanced the TGF-*β*, ANP, and BNP expression in the AB+AAV9-STAT3 group compared with the AB+AAV9-GFP group (Figures 7(h) and 7(i)). Moreover, CTS treatment could not decrease phosphorylation of STAT3 and SMAD3 and alleviate the TGF-*β* and ANP expression in the AB+AAV9-STAT3+CTS group compared with the AB+AAV9-STAT3+VEH group (Figures 7(h) and 7(i)). These experiments demonstrated that CTS could not prevent mouse hearts from pressure overload-induced cardiac remodeling after AAV9-mediated STAT3 overexpression in a mouse heart.

## 4. Discussion

This study demonstrated firstly that CTS could protect against pressure overload-induced mouse cardiac remodeling *in vivo* and prevent the AngII-induced cardiomyocyte hypertrophy and TGF-*β*-induced CF activation *in vitro*. The underling mechanism might be associated with inhibiting STAT3 phosphorylation and its nuclear translocation. Additionally, this study also firstly indicated that CTS could no longer protect mouse heart from pressure overload-induced cardiac remodeling in AAV9-mediated STAT3 overexpression mouse heart.

CTS is a liposoluble and bioactive compound extracted from danshen [3]. Danshen has a long history for treating chest pain, which indicated a syndrome of cardiovascular diseases containing atherosclerosis, angina, and obstructed coronary heart disease [3, 25]. Fufang Danshen Dripping pill has been widely used for mitigating angina pectoris in clinical practice in China [3, 25], especially in these patients who could not tolerate the treatment of nitrates for alleviating angina pectoris [3, 25]. Fufang Danshen Dripping pill is also the first Chinese traditional medicine to pass Phase II trials of the US Food and Drug Administration [3, 25]. A total of 201 chemical constituents have been isolated and differentiated from danshen [3]. CTS is one of the high content representative ingredients in danshen [3]. Studies have exhibited that CTS exerted excellent effects in anti-inflammation, anticancer, and antibacterial [26]. Some studies have also implied that CTS could effectively improve experimental cardiovascular disease [10, 12, 27]. However, it has not been reported whether CTS treatment could directly alleviate pressure overload or neurohumoral stimuli induced pathological remodeling *in vivo* and *in vitro*.

This study firstly reported that CTS treatment could attenuate mouse cardiac hypertrophy and fibrosis. Mechanistically, CTS treatment showed significantly inhibited STAT3 phosphorylation and its nuclear translocation but exhibited nonsignificant effect for JAK1/2 phosphorylation and inhibition of PI3K/AKT and MAPK signaling pathways. STAT3 has been suggested to be an attractive therapeutic target for treating cardiac remodeling and heart failure. Moreover, both pressure overload and angiotensin II could induce JAK phosphorylation, which consequently phosphorylate the STAT3 resulting in accumulation and translocation in the nucleus [28]. By interacting with the promotor domain of downstream target genes in the nucleus, phosphorylated STAT3 could enhance expression of hypertrophic genes such as atrial natriuretic peptide A (NPPA) and Myosin Heavy Chain 7 (MYH7) [29]; thus, STAT3 overexpression in mouse heart caused idiopathic myocardial hypertrophy at 12 weeks of age [29]. Cardiac specific *β*_IV_-spectrin knockout could lead to STAT3 hyperphosphorylation and nuclear translocation resulting in cardiac fibrosis and dysfunction in both baseline and pressure overload mouse heart [30]; however, inhibiting STAT3 could effectively restore cardiac function and structure in *β*_IV_-spectrin knockout and wild-type mouse subjected to pressure overload surgery [30]. STAT3 could be significantly activated by interleukin- (IL-) 6 and Ca (2+)/calmodulin-dependent protein kinase II- (CaMKII-) mediated phosphorylation resulting in exaggerated pressure overload-induced mouse cardiac hypertrophy [31]. These findings have indicated that STAT3 inhibition has markedly therapeutic effect for cardiac hypertrophy and heart failure. This study implied that CTS might directly inhibit STAT3 phosphorylation for attenuating cardiac hypertrophy.

STAT3 has also been demonstrated to be directly involved in regulating myocardial fibrosis, which is characterized by myofibroblast activation and accumulation of extracellular matrix (ECM). Myocardial fibrosis has been suggested to be a common feature of cardiac remodeling at the early stage before cardiac dysfunction and heart failure regardless of etiology of heart diseases. The TGF*β*/SMAD3 signaling pathway has been demonstrated to be the most important signaling pathway involved in cardiac fibrosis; however, direct inhibition of SMAD3 has been demonstrated to not resolve cardiac fibrosis but caused some other side effects. But SMAD3 deficiency reduced 60% of pressure overload-induced myocardial fibrosis while significantly exaggerating cardiac hypertrophy [32]. And SMAD3 deficiency in myofibroblasts significantly promoted MMP-8 secretion resulting in exacerbated pressure overload-induced cardiac remodeling and dysfunction [33]. These might be partly because Smad3 inhibition only limited the proliferation of cardiac fibroblasts and showed no effects for inhibiting the phenotypic transition of fibroblasts to myofibroblasts [34]. Therefore, it is necessary to search new transcription factors or treatment targets for preventing myocardial fibrosis.

STAT3 has been suggested to be another important transcription factor involving in cardiac fibrosis. STAT3 could interact with SMAD3 to promote the differentiation of cardiac fibroblasts into myofibroblasts in hypoxic conditions via enhancing the typical TGF*β*/SMAD3 signaling pathway [35]. TGF-*β* treatment could induce overproduction of STAT3 and collagen in cultured rat atrial fibroblasts [36]. TGF-*β* transgenic mice and atrial fibrillation (AF) patients presented a significant higher expression of STAT3 and collagen in atria than wild-type mice and sinus rhythm subjects, respectively [36]. Mechanistically, chromatin immunoprecipitation exhibited that STAT3 could interact with collagen promoter for enhancing collagen expression [36]. STAT3 phosphorylation at Tyr705 could enhance intermittent hypoxia-induced thrombospondin-1 expression resulting in exacerbated cardiac fibrosis. STAT3 inhibitor S3i-201 or silence of STAT3 in cardiac fibroblast could significantly attenuate IH- or AngII-induced cardiac fibrosis in mice [37]. These studies indicated that STAT3 inhibition might be a potential strategy for blocking cardiac fibrosis. This study demonstrated that CTS treatment could attenuate pressure overload induced mouse cardiac fibrosis *in vivo* and prevent AngII-induced myocardial fibroblast activation *in vitro* via inhibiting STAT3 phosphorylation and nuclear translocation. A previous study also exhibited CTS treatment could attenuate cardiac fibrosis in type 1-like diabetic rats via suppressing STAT3 pathway [10]. In addition to inhibiting myocardial fibrosis, CTS treatment could attenuate lung injuries and decrease ECM deposition in a bleomycin-induced pulmonary fibrosis model [8]. Mechanistically, CTS treatment could significantly inhibit Smad2/3 and STAT3 phosphorylation in TGF-*β* treated human fetal lung fibroblasts (HFLs) [8]. STAT3 overexpression partially neutralized the treatment effect of CTS in preventing TGF-*β*-induced fibrosis response in HFLs [8]. This investigation along with previous studies exactly demonstrated that CTS might be a potential medication or adjuvant medication for preventing cardiac or lung fibrosis via inhibiting STAT3-dependent signaling pathway.

## 5. Conclusions

This study concluded that CTS could be a potential treatment or adjuvant treatment for protect against pressure overload or neurohumoral stimuli-induced cardiac remodeling. The underlying mechanism might at least partly be associated with inhibiting STAT3 signaling pathway. Although CTS exhibited powerful function for blocking cardiac remodeling in this preliminary investigation via inhibiting STAT3 dependent signaling pathway, some investigations were necessarily furtherly clarifying the potential adverse effects and some other potential mechanisms. This study and previous studies have demonstrated that CTS-mediated STAT3 inhibition might be advantageous for preventing pressure overload or AngII-induced cardiac remodeling; however, some published researches showed that STAT3 inhibition might be detrimental in the heart. Leukemia inhibitory factor (LIF) or adenovirus-mediated constitutively activation of STAT3 (CaSTAT3) could block hypoxia/reoxygenation- (H/R-) induced release of creatine phosphokinase and protect against H/R-associated cardiomyocyte injury by inhibiting ROS generation [38]. In addition, CaSTAT3 could protect against ischemia/reperfusion induced injury via STAT3-mediated upregulation of metallothionein1/2 [39]. These findings indicated that STAT3 activation might exert a protective role in response to I/R and HO via reducing oxidative stress. It remained unclear whether sustained CTS treatment could disturb the balance of oxidative stress in pressure overload or AngII-induced cardiac remodeling. Besides, CTS has exhibited multiple roles in regulating different signal pathways and biological function in several other disease models [3]; it will be better to evaluate its biological roles and mechanisms via transcriptome and metabolomics in future experiments. We believed that it will be very interesting and necessary to further study the roles and mechanisms of CTS in different models of cardiac remodeling, because Fufang Danshen Dripping pill (containing CTS) has been widely used for treating heart disease in clinical practice.

## Figures and Tables

**Table 1:** Primers used in this study.

Species	Gene name	Forward primer	Reverse prime
Mouse	GAPDH	ACTCCACTCACGGCAAATTC	TCTCCATGGTGGTGAAGACA
Mouse	NPPA	ATTGACAGGATTGGAGCCCAG	TCAAGCAGAATCGACTGCCTT
Mouse	NPPB	TTTGGGCTGTAACGCACTGA	CACTTCAAAGGTGGTCCCAGA
Mouse	MYH7	CCGAGTCCCAGGTCAACAA	CTTCACGGGCACCCTTGGA
Mouse	Tgfb	ATCCTGTCCAAACTAAGGCTCG	ACCTCTTTAGCATAGTAGTCCGC
Mouse	CCN2	AGACCTGTGCCTGCCATTAC	ACGCCATGTCTCCGTACATC
Mouse	COL1A1	AGCACGTCTGGTTTGGAGAG	GACATTAGGCGCAGGAAGGT
Mouse	COL3A1	TGACTGTCCCACGTAAGCAC	GAGGGCCATAGCTGAACTGA
Rat	GAPDH	GACATGCCGCCTGGAGAAAC	AGCCCAGGATGCCCTTTAGT
Rat	CCN2	GGAAGACACATTTGGCCCTG	GCAATTTTAGGCGTCCGGAT
Rat	COL1A1	GAGAGAGCATGACCGATGGATT	TGGACATTAGGCGCAGGAA
Rat	COL3A1	AAGGGCAGGGAACAACTGAT	GTGAAGCAGGGTGAGAAGAAAC

## Data Availability

Data will be available upon request from the corresponding author.

## Supplementary Materials

Supplementary Materials Supplementary figure 1: CTS treatment showed none significant effect on JAK, MAPKs and PI3K/AKT pathways. (A) Representative western-blots of p-JAK1, T-JAK 1, p-JAK 2, T-JAK 2, and GAPDH (*n* = 6); relative quantification of (B) p-JAK/T-JAK 1 and (C) p-JAK 2/T-JAK 2; (D) representative western-blots of p-P38, T-P38, p-ERK1/2, T-ERK1/2, p-JNK1/2, T-JNK1/2, and GAPDH (*n* = 6); (E) representative western-blots of p-PI3K, T-PI3K, p-AKT, T-AKT, and GAPDH (*n* = 6); and (F) relative quantification of p-P38/T-P38, p-ERK1/2/T-ERK1/2, p-JNK1/2/T-JNK1/2, p-PI3K/T-PI3K, and p-JNK1/2/T-JNK1/2 (*n* = 6); all of these proteins were normalized to GAPDH before the relative quantification. ^∗^Compared with the VE+Sham group (*p* < 0.05); ns: nonsignificant difference. Supplementary figure 2: AngII treatment induced STAT3 hyper-phosphorylation. (A) Representative Western blots of p-STAT3, T-STAT3, and GAPDH and NRCMs were incubated with AngII for indicated times; (B) relative quantification of p-STAT3/T-STAT3. Phosphorylated protein was normalized to corresponding total protein and then normalized to GAPDH before relative quantification; cell experiments were repeated three times independently. ^∗^Compared with the PBS-treated group (*p* < 0.05); ^#^compared with the AngII-treated group (*p* < 0.05). Supplementary figure 3: CTS treatment did not improve mouse cardiac function after AAV-9 mediated STAT3 overexpression (A) HR, (B) LVEF, (C) FS, and (D) LVEDs. Data presented as mean ± S.E.; ^∗^compared with the AAV9-GFP group (*p* < 0.05); ^#^compared with the AAV9-GFP group+AB group (*p* < 0.05).
