# Supplementary material for: Cryptotanshinone Attenuated Pathological Cardiac Remodeling In Vivo and In Vitro Experiments
Source: Oxid Med Cell Longev. 2023 Jan 27;2023:4015199. doi: 10.1155/2023/4015199 (PMC9897919; doi:10.1155/2023/4015199)
Supplement: Supplementary Materials — Supplementary figure 1: CTS treatment showed none significant effect on JAK, MAPKs and PI3K/AKT pathways. (A) Representative western-blots of p-JAK1, T-JAK 1, p-JAK 2, T-JAK 2, and GAPDH (n = 6); relative quantification of (B) p-JAK/T-JAK 1 and (C) p-JAK 2/T-JAK 2; (D) representative western-blots of p-P38, T-P38, p-ERK1/2, T-ERK1/2, p-JNK1/2, T-JNK1/2, and GAPDH (n = 6); (E) representative western-blots of p-PI3K, T-PI3K, p-AKT, T-AKT, and GAPDH (n = 6); and (F) relative quantification of p-P38/T-P38, p-ERK1/2/T-ERK1/2, p-JNK1/2/T-JNK1/2, p-PI3K/T-PI3K, and p-JNK1/2/T-JNK1/2 (n = 6); all of these proteins were normalized to GAPDH before the relative quantification. ∗Compared with the VE+Sham group (p < 0.05); ns: nonsignificant difference. Supplementary figure 2: AngII treatment induced STAT3 hyper-phosphorylation. (A) Representative Western blots of p-STAT3, T-STAT3, and GAPDH and NRCMs were incubated with AngII for indicated times; (B) relative quantification of p-STAT3/T-STAT3. Phosphorylated protein was normalized to corresponding total protein and then normalized to GAPDH before relative quantification; cell experiments were repeated three times independently. ∗Compared with the PBS-treated group (p < 0.05); #compared with the AngII-treated group (p < 0.05). Supplementary figure 3: CTS treatment did not improve mouse cardiac function after AAV-9 mediated STAT3 overexpression (A) HR, (B) LVEF, (C) FS, and (D) LVEDs. Data presented as mean ± S.E.; ∗compared with the AAV9-GFP group (p < 0.05); #compared with the AAV9-GFP group+AB group (p < 0.05). [file 4015199.f1.docx]

**Supplementary Material**

Supplementary Figures

**
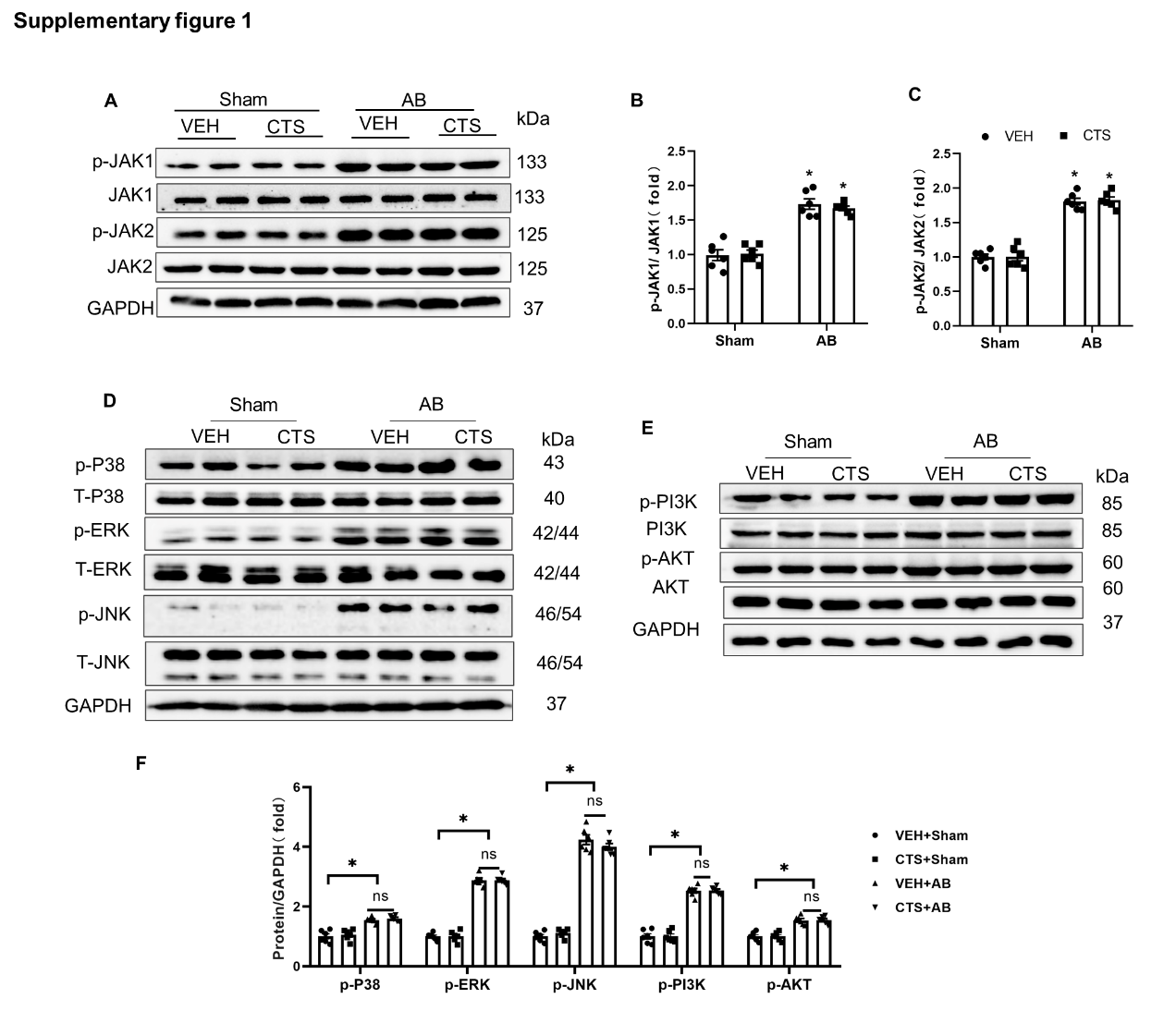
**

**Supplementary figure 1 CTS treatment showed none significant effect on JAK, MAPKs and PI3K/AKT pathways** (A) Representative western-blots of p-JAK1, T- JAK 1, p- JAK 2, T- JAK 2 and GAPDH (n=6), relative quantification of (B) p- JAK /T- JAK 1, (C) p- JAK 2/T- JAK 2 (D) Representative western-blots of p-P38, T-P38, p-ERK1/2, T-ERK1/2, p-JNK1/2, T-JNK1/2 and GAPDH (n=6) (E) Representative western-blots of p-PI3K, T-PI3K, p-AKT, T-AKT and GAPDH (n=6) (F) Relative quantification of p-P38/T-P38, p-ERK1/2/T-ERK1/2, p-JNK1/2 /T-JNK1/2, p-PI3K/T-PI3K and p-JNK1/2 /T-JNK1/2 (n=6), all of these proteins were normalized to GAPDH before the relative quantification, * compared with VE+Sham group *p* < 0.05, ns: none significant difference.


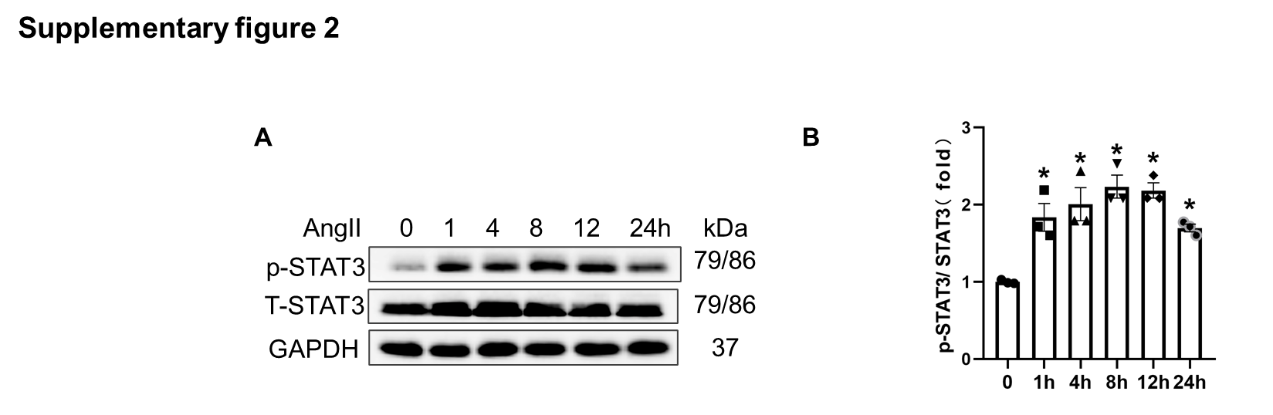


**Supplementary figure 2 AngII treatment induced STAT3 hyper-phosphorylation**

1. Representative western-blots of p-STAT3, T-STAT3 and GAPDH, NRCMs were incubated with AngII for indicated times, (B) relative quantification of p-STAT3/T-STAT3. phosphorylated protein was normalized to corresponding total protein and then normalized to GAPDH before relative quantification, cell experiments were repeated three times independently. * compared with PBS treated group *p* < 0.05, # compared with AngII treated group *p* < 0.05.


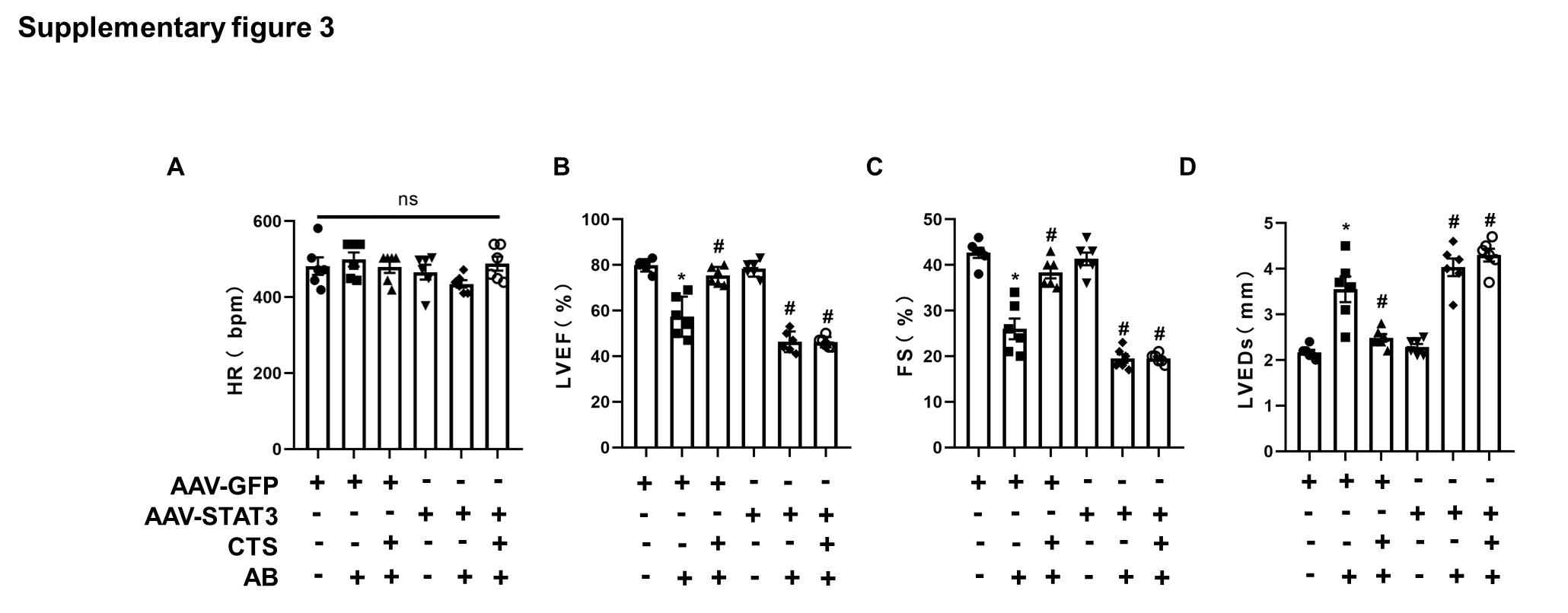


**Supplementary figure 3 CTS treatment didn’t improve mouse cardiac function after AAV-9 mediated STAT3 overexpression** (A) HR, (B) LVEF (C) FS, (D) LVEDs Data presented as mean ± S.E., * compared with AAV9-GFP group *p* < 0.05, # compared with AAV9-GFP group+ AB group *p* < 0.05.
